# Protocol for a Randomized, Open-Label Clinical Trial on the Effect of Mouthwash on Salivary SARS-CoV-2 Load

**DOI:** 10.3390/life13122312

**Published:** 2023-12-08

**Authors:** Keiji Konishi, Daisuke Onozuka, Satoko Takatera, Hiroo Matsuo, Hisao Yoshida, Shigeto Hamaguchi, Shungo Yamamoto, Ryuichi Minoda Sada, Koichiro Suzuki, Satoshi Kutsuna

**Affiliations:** 1 Department of Oral Microbe Control, Graduate School of Medicine, Osaka University, Osaka 565-0871, Japan; onozukad@hp-infect.med.osaka-u.ac.jp (D.O.); s.takatera@hp-infect.med.osaka-u.ac.jp (S.T.); 2 Department of Infection Control and Prevention, Graduate School of Medicine, Osaka University, Osaka 565-0871, Japan; matsuo@hp-infect.med.osaka-u.ac.jp (H.M.); h-yoshida@yuikuen.com (H.Y.); hamaguchi@hp-infect.med.osaka-u.ac.jp (S.H.); shungo1977@cider.osaka-u.ac.jp (S.Y.); sadao@cider.osaka-u.ac.jp (R.M.S.); 3 Department of Transformative Analysis for Human Specimen, Graduate School of Medicine, Osaka University, Osaka 565-0871, Japan; 4 Division of Fostering Required Medical Human Resources, Center for Infectious Disease Education and Research (CiDER), Osaka University, Osaka 565-0871, Japan; 5 Department of Transformative Protection to Infectious Disease, Graduate School of Medicine, Osaka University, Osaka 565-0871, Japan; 6 The Research Foundation for Microbial Diseases of Osaka University (BIKEN), Osaka 565-0871, Japan; ksuzuki@mail.biken.or.jp

**Keywords:** cetylpyridinium chloride, COVID-19, SARS-CoV-2, mouthwash, on-demand aqueous chlorine dioxide solution, open-label randomized clinical trial

## Abstract

Mouthwashes containing cetylpyridinium chloride (CPC) or on-demand aqueous chlorine dioxide (ACD) have potential to reduce the salivary severe acute respiratory syndrome coronavirus 2 (SARS-CoV-2) load in individuals with SARS-CoV-2 infection. This study will evaluate the effect of CPC and on-demand ACD mouthwashes on salivary SARS-CoV-2 levels in individuals with acute asymptomatic or mild SARS-CoV-2 infection (COVID-19) staying in a residential recuperation facility in Osaka, Japan. This randomized, open-label clinical trial will include three equal-sized groups (CPC mouthwash, on-demand ACD mouthwash, and placebo), with 30 participants per group. A stratified replacement block method will be used to ensure balanced allocation based on symptom presence and days since symptom onset. Participants will use mouthwash at set times for 7 days or until the end of recuperation. Saliva samples will be collected at multiple time points and tested for SARS-CoV-2 using quantitative reverse transcription polymerase chain reaction. The primary outcome will be changes in salivary SARS-CoV-2 viral load 2 h after the first mouthwash use compared with the pre-mouthwash level. Secondary outcomes will include changes in salivary viral load and clinical parameters at different time points. This study was registered with the Japan Registry of Clinical Trials on 18 October 2022 (jRCTs051220107).

## 1. Introduction

Ever since the abrupt emergence of severe acute respiratory syndrome coronavirus 2 (SARS-CoV-2) in the People’s Republic of China, coronavirus disease 2019 (COVID-19) has left an indelible global footprint. This viral scourge has resulted in substantial morbidity, mortality, and significant economic ramifications.

COVID-19 infection involves the oral cavity, suggesting that saliva and the salivary glands potentially serve as sources for the transmission of the virus. Consequently, there has been a focus on investigating mouthwashes containing substances that exhibit virucidal activity, with the aim of preventing COVID-19 infection and mitigating the spread of the virus. Cetylpyridinium chloride (CPC) is a bactericide frequently used in mouthwashes, lozenges, and sprays [1]. In vitro studies have shown that CPC inactivates SARS-CoV-2 [2,3]. Because CPC disrupts lipid bilayers, high concentrations of CPC can have cytotoxic effects, but even low concentrations of CPC (0.001–0.005%) have been reported to inhibit the infectivity of SARS-CoV-2 Wuhan and the Alpha, Beta, and Gamma variants [1]. The antiviral effect is due to the denaturation of SARS-CoV-2 protein, rather than the disruption of lipid membranes [1]. Previous studies are limited by their small sample sizes. In a clinical study conducted in Singapore on patients with COVID-19, a reduction in the SARS-CoV-2 salivary viral load was observed 5 min and 6 h after using a CPC-containing mouthwash (four participants) compared with a control group (two participants) [4]. Although the sample size was small, CPC reduced the salivary SARS-CoV-2 viral load [5].

A novel mouthwash, MA-T^TM^ (developed by the Japan MA-T Industrial Association, Tokyo, Japan), was introduced to address the shortcomings of conventional chlorine dioxide solutions intended for human use. This mouthwash featured an on-demand aqueous chlorine dioxide (ACD) formulation [6]. In vitro studies have shown that chlorine dioxide and on-demand ACD have strong antiviral effects against SARS-CoV-2 [7], but no clinical studies have been conducted to date.

Based on previous clinical investigations, it is anticipated that CPC may attenuate the presence of SARS-CoV-2 in salivary specimens. However, this requires confirmation owing to the limited sample size in previous studies. The primary objective of this study is to assess the efficacy of CPC and on-demand ACD mouthwashes compared with a placebo at lowering the SARS-CoV-2 viral load in the saliva of individuals with mild or asymptomatic SARS-CoV-2 infection. If this investigation confirms the efficacy of mouthwashes containing CPC and on-demand ACD in mitigating SARS-CoV-2 levels in saliva, this could make a useful contribution to the prophylaxis of SARS-CoV-2 infection, and to transmission, especially in group settings.

Participant enrollment started in October 2022 and was completed in January 2023. As of October 2023, data analysis is in progress.

## 2. Experimental Design

This protocol was written in accordance with the Standard Protocol Items: Recommendations for Interventional Trials (SPIRIT) reporting template [8] (Supplementary Materials S1 file). This single-center, randomized (1:1:1), open-label clinical trial has three treatment groups: CPC mouthwash, on-demand ACD mouthwash, and placebo. A stratified replacement block method will be used for allocation. Allocation factors include the presence or absence of symptoms and the number of days elapsed since their onset (asymptomatic, within 3 days of onset, or ≥4 days since onset). All three groups will use a mouthwash or placebo from day 1 to day 7, until the last recuperation day or treatment discontinuation, whichever comes first.

### 2.1. Study Setting

This study will include patients with mild or asymptomatic SARS-CoV-2 infection who are recuperating at a specific hotel designated for COVID-19 treatment by the Osaka Prefecture health authority.

### 2.2. Eligibility Criteria

#### 2.2.1. Inclusion Criteria

- Age ≥ 18 years;
- A confirmed COVID-19 diagnosis using nucleic acid amplification tests (reverse transcription polymerase chain reaction (RT-PCR), loop-mediated isothermal amplification (LAMP), or antigen tests), with or without mild COVID-19 symptoms ≤7 days after onset;
- Recuperating at a designated hotel in Osaka, Japan;
- No clinical contraindications for mouthwash use;
- Smartphone access with communication applications.

#### 2.2.2. Exclusion Criteria

- A previously confirmed diagnosis of COVID-19, with or without hospitalization;
- An incipient need for hospitalization;
- Currently pregnant/breastfeeding;
- Receipt of antiviral or immunosuppressive medicines since the onset of COVID-19, including remdesivir, molnupiravir, nirmatrelvir/ritonavir, sotrovimab, casirivimab/imdevimab, anti-interleukin-6 receptor antibody, Janus kinase inhibitors, or corticosteroids.

### 2.3. Objectives

The primary objective of this study is to assess the efficacy of CPC and on-demand ACD mouthwashes compared with a placebo at lowering the SARS-CoV-2 viral load in the saliva of individuals with mild or asymptomatic SARS-CoV-2 infection.

#### 2.3.1. Primary Outcome

The primary outcome is the change in salivary SARS-CoV-2 viral load 2 h after the initial mouthwash application compared with that before the first mouthwash application.

#### 2.3.2. Secondary Outcomes

The secondary outcomes are:

1. The change in salivary SARS-CoV-2 viral load 30 min after the initial mouthwash application compared with the baseline measurement;
2. The change in salivary SARS-CoV-2 viral load 4 h after the initial mouthwash application compared with the baseline measurement;
3. The change in salivary SARS-CoV-2 viral load 10 h after the initial mouthwash application compared with the baseline measurement;
4. The change in salivary SARS-CoV-2 viral load 24 h after the first mouthwash application compared with the baseline measurement;
5. The change in transcutaneous arterial blood oxygen saturation (SpO_2_) 24 h after the first mouthwash application (measured on the morning of day 2) compared with the baseline measurement;
6. The change in the clinical condition rating scale 24 h after the first mouthwash application (measured on the morning of day 2) compared with the baseline measurement;
7. The change in SpO_2_ 7 days after the first mouthwash application (measured on the morning of day 7) or on the last recuperation day, compared with the baseline measurement;
8. The change in the clinical condition rating scale 6 days after the first mouthwash application (measured on the morning of day 7 of mouthwash use) or on the last recuperation day, compared with that before the first mouthwash application.

### 2.4. Sample Size

A parallel, 3-group design will be used. The hypotheses will be evaluated using two-sided, two-sample, Bonferroni-adjusted, unequal-variance (Welch’s) *t*-tests, with an overall Type I error rate (α) of 0.05. The group standard deviations (beginning with the control group) will be assumed to be 1, 1, and 1. The control group mean will be assumed to be 0. To detect a mean reduction in salivary viral load of 90% in participants using mouthwash, with at least 80% power for each test, the sample size needed for each of the three groups (calculated using PASS 2022) is 25. Considering the feasibility and the possibility of some participants not completing the intervention, the target sample size is 30 cases per group.

### 2.5. Statistical Methods

Where applicable, the baseline participant characteristics will be presented as frequencies and proportions (%) for categorical data and the median and interquartile range (IQR) for continuous data. Linear mixed-effects models will be used to evaluate the effect of the CPC and ACD mouthwashes on salivary SARS-CoV-2 viral load compared with a placebo. As the study interventions are considered low-risk interventions, no interim analyses are planned. No subgroup analyses or additional analyses are planned. The statistical tests will be two-tailed and a value of *p* < 0.05 will be considered to indicate statistical significance. All analyses will be performed using Stata 18.0 (StataCorp LLC, College Station, TX, USA).

### 2.6. Materials and Equipment

The CPC and on-demand ACD mouthwashes will be manufactured by the Earth Corporation (Tokyo, Japan), and the placebo will be obtained from the Hikari Pharmaceutical Corporation (Tokyo, Japan). The composition and characteristics of each test product are shown in Table 1.

CPC mouthwash contains CPC, dipotassium glycyrrhizate (GK2), and tranexamic acid (TXA) as active ingredients and is effective in preventing gingivitis, bleeding, plaque buildup, and halitosis and in freshening the mouth. The other ingredients are shown in Table 1. CPC, a bactericidal ingredient, is known to have bactericidal effects on bacteria and fungi and is widely used in pharmaceuticals and quasi-drugs in the dental field, owing to its bactericidal effects on various oral bacteria. GK2 is also recognized to have an anti-inflammatory effect, and TXA prevents bleeding from the gums owing to gingivitis.

On-demand ACD is a mouthwash with effects leading to the prevention of halitosis and purification of the mouth; as of 3 March 2021, it has been reported to the Tokyo Metropolitan Government as an oral cosmetic product and is already commercially available.

On-demand ACD is a time-produced aqueous chlorite ion solution, wherein chlorite ions react with catalytic components to generate aqueous radicals; this product is based on the Matching Transformation System (MA-T), an oxidation control system developed by AceNet Co. The main component of on-demand ACD is water, but it also contains 50 ppm of MA-T (time-produced aqueous chlorite ion solution). The other ingredients are shown in Table 1. Various safety tests have been conducted on MA-T (time-produced aqueous chlorite ion solution); experiments using a human oral epithelial cell model have shown that MA-T at a concentration of 500 ppm causes no irritation and is a safe product.

### 2.7. Recruitment

Approximately 10 patients with COVID-19 are admitted to the recuperation hotel in Osaka daily. Recruitment is expected to be completed without difficulty.

### 2.8. Implementation

The principal investigator or subinvestigator will enter the necessary information into the web-based allocation system for the patients who are determined to be eligible to participate, and the web-based allocation system will assign the participant to a mouthwash/placebo group.

## 3. Procedures

### 3.1. Informed Consent

One of the investigators will meet with the patient online to explain the research procedures and obtain informed consent. During the consent process, participants will be asked about their willingness to allow the use of their data in the event of their withdrawal from this study. Because this is a single-center trial, the consent form will not include a section regarding data-sharing permission. All requisite measures will be implemented to safeguard the privacy and confidentiality of participants. Participants will be provided with information on how to contact the study team to facilitate inquiries and to address any concerns pertaining to study participation.

### 3.2. Interventions

Three groups will be included. The two intervention groups will use either 20 mL of mouthwash containing 0.05% CPC for 30 s or 10 mL of mouthwash containing 0.01% ACD on-demand for 1 min, depending on their group assignment, and the control group will use 20 mL of placebo (purified water) for 1 min. All other medical interventions will be identical between groups. Purified water was chosen as the comparator because it does not have antiviral activity and water is generally used as a mouthwash when brushing the teeth.

#### Mouthwash Application Method

Eligible patients will be randomly assigned to groups and will be instructed to use specific mouth rinses as follows: a 20 mL placebo solution (consisting of purified water) for 1 min, a 20 mL mouthwash containing 0.05% CPC for 30 s, or a 10 mL mouthwash with 0.01% on-demand ACD for 1 min. This regimen will be followed for 7 days. A baseline saliva sample will be collected before initiating the mouthwash regimen. Following the sample collection, patients will rinse their mouths with the assigned mouthwash or placebo. Six saliva samples will be collected at the following time points: baseline (immediately before breakfast at 08:00), 30 min after baseline (08:30), 2 h after baseline (10:00), 4 h after baseline (immediately before lunch at 12:00), 10 h after baseline (immediately before dinner at 18:00), and 24 h after baseline (immediately before breakfast the following morning at 08:00).

After each saliva sample collection, participants will be instructed to rinse their mouths with the allocated mouthwash or water at baseline and again at the 4, 10, and 24 h time points. The saliva samples will be individually collected in sterile tubes at the hotel, inactivated, and stored at 4 °C in a refrigerator at Osaka University. The saliva samples will then be sent to the Research Foundation for Microbial Diseases of Osaka University (BIKEN), and RT-PCR testing for SARS-CoV-2 will be conducted within 36 h of sample collection. Saliva collected according to this research protocol will be stored in a freezer at −80 °C in the laboratory of Infection Control, Osaka University Graduate School of Medicine for one year after study completion.

### 3.3. Assignment of Interventions: Allocation

Participants will be individually assigned to one of the three groups in a 1:1:1 ratio. A stratified substitution block method will be used for allocation, in which the presence or absence of symptoms and the number of days elapsed since onset (asymptomatic, within 3 days of onset, or 4 days or more since onset) are used as allocation factors. One of the investigators will enter the necessary information into the web-based allocation system for the patients who are determined to be eligible to participate, and the web-based allocation system will assign the participant to a mouthwash/placebo group. This is an open-label trial. Neither the participants nor the investigators will be blinded to the intervention assignment.

### 3.4. Criteria for Discontinuing or Modifying Allocated Interventions

Participants will be free to leave this study at any time for any reason without consequences. The investigator may withdraw participants from this study for medical reasons. These may include any of the following:

1. If symptoms such as abnormalities in the mouth, a rash, or itching occur after using the mouthwash;
2. If an adverse event occurs and the principal investigator or co-investigator determines that there is an unacceptable risk to the health of the research participants if this study is continued;
3. If the participant requests to withdraw;
4. If the participant is discharged from the residential care facility prior to the saliva collection on the second day of mouthwash (immediately before the morning mouthwash) due to a worsening of the participant’s condition;
5. If COVID-19 therapeutic drug administration or respiratory therapy becomes necessary due to COVID-19 or comorbidities;
6. If the participant is found to be unsuitable or ineligible for participation;
7. If it is not possible to conduct necessary observations or examinations at the convenience of the participant;
8. Other cases in which the investigators determine that the intervention should be discontinued for any reason.

### 3.5. Strategies to Improve Adherence to Interventions

Online interviews will be conducted on days 2 and 7 of mouthwash use to confirm adherence to the intervention.

### 3.6. Relevant Concomitant Care Permitted or Prohibited during the Trial

1. Concomitant medications and therapies for comorbidities will not be restricted.
2. Participants will be asked not to use toothpaste containing ingredients with antiviral activity until after the morning saliva collection on the second day of mouthwash use. The participants will be given toothpaste that does not contain ingredients with antiviral activity to use if they brush their teeth before the end of saliva collection in the morning on the second day of mouthwash use. Additionally, participants will be instructed not to brush their teeth excessively while participating in this study.
3. Participants will be asked to avoid taking CPC-containing throat lozenges from the time that they wake up on the first day of mouthwash use until after the morning saliva collection on the second day of mouthwash use.
4. Participants will also be asked to avoid drinking tea or eating foods containing plums or persimmon tannin immediately prior to saliva collection.
5. Participants will be instructed to refrain as much as possible from drinking water for 30 min prior to saliva collection.

### 3.7. Participant Timeline

Table 2 shows the participant timeline.

### 3.8. Plans to Promote Participant Retention and Complete Follow-Up

After enrollment and randomization, the investigators will make every reasonable effort to follow the participant throughout the entire study period. Participants will be free to discontinue their study participation at any time. Participant retention will be encouraged by conducting online interviews.

## 4. Data Collection and Management

### 4.1. Plans for Assessment and Collection of Outcomes

The timing of outcome assessments is shown in Table 1. Saliva will be collected for SARS-CoV-2 viral load measurement at baseline and 30 min, 2 h, 4 h, 10 h, and 24 h after first mouthwash application. SpO_2_ will be measured in the morning at baseline and on days 2 and 7 of mouthwash use. If mouthwash use is discontinued within 7 days, SpO_2_ will be measured in the morning of the day of discontinuation instead of on day 7. The clinical condition rating scale will be administered at baseline and on days 2 and 7 of mouthwash use. If mouthwash use is discontinued within 7 days, the clinical condition rating scale will be administered on the day of discontinuation instead of on day 7.

### 4.2. Data Management

Data will be collected on participant sex, age, body mass index, smoking history, comorbidities, vaccination status, interval between symptom onset and diagnosis, and oxygen saturation (SpO_2_). The data obtained will be kept until five years after the completion of this study.

### 4.3. Confidentiality

The confidentiality of participants will be maintained. The patients will be identified on case report forms (CRFs) and other documents by their age and identification number throughout this study. Records that contain personal identifiers (e.g., the signed informed consent form) will be maintained securely by the investigators. Participants will be informed that all their study data will be stored on a password-protected computer and that confidentiality will be maintained.

## 5. Oversight and Monitoring

### 5.1. Ethics Approval

This study will be conducted in accordance with the Declaration of Helsinki and has been approved by the Osaka University Ethics Committee (protocol code S22003 and approved October 2022).

#### 5.1.1. Composition of the Coordinating Center and Trial Steering Committee

This is an investigator-initiated, single-center trial, which is run by a small study team. There is no coordinating center or trial steering committee. Organizational support will be provided by the Medical Center for Translational Research at the Osaka University Hospital. The study team will meet daily to review routine clinical cases, and study-related topics will be discussed during this daily meeting as appropriate. The Medical Center for Translational Research at the Osaka University Hospital will consult during the study initiation and closure phases, as well as on request.

#### 5.1.2. Composition of the Data Monitoring Committee, its Role, and the Reporting Structure

In order to ensure that the clinical research is properly conducted from the perspective of ensuring its credibility and protecting research participants, the principal investigator will designate a person who will be in charge of monitoring the progress of the research, monitoring whether the research is being conducted in accordance with the regulations and research protocol, and preparing a monitoring protocol; the same person will conduct monitoring in accordance with the monitoring protocol and prepare a record of the monitoring (monitoring report).

### 5.2. Adverse Event and Harm Reporting 

Mouthwash is a safe product approved in Japan for over-the-counter purchase without a prescription, and it is unlikely to cause obvious side effects, even with long-term use. If a serious adverse event is suspected to be related to this study, the investigators may temporarily suspend the participant’s participation in this study. An adverse event is any unwanted or unintended sign (including abnormal laboratory values), symptom, or illness in a research participant, regardless of whether it is causally related to the study intervention. Adverse event collection will begin at the time of the first mouthwash administration and will end after the last mouthwash administration. The sponsor and principal investigator will initiate the suspension of this study in the presence of credible concerns indicating a potential compromise to the safety and wellbeing of participants. The Osaka University Ethics Committee will be notified of any concerns without delay, and this study will remain in suspension until a decision is reached by the local ethics committee regarding its resumption. All reported adverse events will be meticulously documented, and any serious adverse events will be reported to the Osaka University Ethics Committee immediately.

### 5.3. Plans for Communicating Important Protocol Amendments to Relevant Parties

Ethical approval of a formal protocol amendment will be required for any significant modification of the study procedures. Participant informed con will be renewed if any protocol changes are made after participants have signed consent, before they have completed the intervention.

### 5.4. Dissemination Plans

Confidentiality of participants’ data will be rigorously upheld in all publications, encompassing presentations at national and international conferences as well as submissions to scientific journals. Ownership of the study data will rest with Osaka University. The trial outcomes will be disseminated by publication in an open-access, peer-reviewed scientific journal (pending determination). There are no restrictions on publication, ensuring that the trial results will be accessible to the public.

### 5.5. Patient and Public Involvement

No patients or members of the general public are involved in protocol development or study implementation. 

## 6. Expected Results

Based on previous clinical study results, it is expected that CPC will reduce the salivary SARS-CoV-2 viral load. However, the number of cases in this study is small; therefore, it may not provide sufficient evidence to draw a definite conclusion. If this study confirms that CPC-containing mouthwash reduces salivary SARS-CoV-2, it may contribute to the COVID-19 infection prevention. If the on-demand ACD mouthwash is found to reduce salivary SARS-CoV-2, ACD mouthwash could be introduced as a new infection prevention measure. The results of this study are expected to provide important information regarding mouthwash’s usefulness in oral hygiene.

This study has several practical implications. Mouthwashes are commonly used in conjunction with brushing teeth as cost-effective and user-friendly disinfectants to maintain oral hygiene [9]. If mouthwash use is confirmed to reduce the presence of SARS-CoV-2 in the oral cavity, its use in populations with a high risk of SARS-CoV-2 infection, including healthcare workers, might be beneficial. It could also be recommended for use in environments in which the risk of infection is high. If this study shows that mouthwashes are effective at preventing SARS-CoV-2 infection, the findings could influence health policies and guidelines. Health authorities and organizations may consider incorporating mouthwash use as part of their recommended COVID-19 prevention measures. In addition, this study’s focus on individuals with asymptomatic or mild COVID-19 provides a targeted intervention among individuals who may not be hospitalized but can still spread the virus to others. If mouthwashes are shown to be effective, they could be recommended as a prevention measure for this specific population. Additionally, their use may assist in situations where infection control is important. Depending on this study’s results, further comprehensive studies and large-scale clinical trials may be warranted. Additionally, publication of this protocol may stimulate the conduct of comparative studies with other mouthwash ingredients.

## 7. Discussion

COVID-19 is a significant public health concern because of its high associated morbidity and mortality. The primary objective of this study is to assess the efficacy of CPC and on-demand ACD mouthwashes compared with a placebo at lowering the SARS-CoV-2 viral load in the saliva of individuals with mild or asymptomatic SARS-CoV-2 infection. This study has several limitations. First, it is a single-center study, and different populations may have varying responses to the interventions. Conducting the trial at a single center may limit the generalizability of the findings. Second, the trial does not use blinding in the study group assignments. Consequently, knowledge regarding the allocation of mouthwash/placebo and the degree of intervention adherence may affect the outcomes. Finally, the intervention duration is short. Although the intervention period is 7 days, a longer follow-up period could provide further insight into the duration of any observed effects.

Overall, mouthwashes containing CPC and ACD might reduce the SARS-CoV-2 load in the oral cavity. If this study confirms that is the case, these mouthwashes could be used as a tool to reduce the risk of spreading infection. Further research and clinical trials will be needed to assess the efficacy and appropriate use of mouthwashes for reducing SARS-CoV-2 transmission.

## Figures and Tables

**Table 1:** Composition and characteristics of each test product.

	CPC Mouthwash	On-Demand ACD Mouthwash
Active ingredients	Cetylpyridinium chloride hydrate, dipotassium glycyrrhizate, tranexamic acid	Water, 50 ppm of time-produced aqueous chlorite ion solution
Additives	Propylene glycol, concentrated glycerin, polyoxyethylene hardened castor oil, sodium dihydrogen phosphate, sodium monohydrogen phosphate, xylitol, paraoxybenzoic acid esters, fragrance, dye	Disodium hydrogen phosphate, sodium hydroxide, benzalkonium chloride, xylitol
Description	Clear pale blue liquid with a peppermint-like aroma and a sweet, refreshing taste	Clear colorless to slightly yellowish liquid
pH	6.1 ± 0.4	7.0 ± 1.0

**Table 2:** Schedule of observation, examination, and evaluation.

	Day of Enrollment	1st Day of Mouthwash Use	2nd Day of Mouthwash Use	3rd to 6th Day of Mouthwash Use or Day before Departure	7th Day of Mouthwash Use or Last Study Day
Obtaining consent	X				
Registration and allocation	X				
Background of study participants	X				
Comorbid disease	X				
Height and weight	X				
SpO_2_ measurement		X	X		X
Saliva collection (just before morning mouthwash)		X	X		
Mouthwash (just before breakfast)		X	X	X	X
Saliva collection (30 min after initial mouthwash)		X			
Saliva collection (2 h after initial mouthwash)		X			
Saliva collection (immediately before the midday mouthwash)		X			
Mouthwash (just before lunch)		X	X	X	X
Saliva collection (immediately before evening mouthwash)		X			
Mouthwash (just before dinner)		X	X	X	X
Online interviews	X		X		X
Clinical condition rating scale	X		X		X
Adverse events		X	X	X	X

## Data Availability

Not applicable.

## Supplementary Materials

The following supporting information can be downloaded at: https://www.mdpi.com/article/10.3390/life13122312/s1, S1 file: Reporting checklist for protocol of a clinical trial.

## Abbreviations

ACD, aqueous chlorine dioxide; COVID-19, coronavirus disease 2019; CPC, cetylpyridinium chloride; jRCT, Japan Registry of Clinical Trials; RT-PCR, reverse transcription polymerase chain reaction; SARS-CoV-2, severe acute respiratory syndrome coronavirus 2.

## References

[1] Takeda R., Sawa H., Sasaki M., Orba Y., Maishi N., Tsumita T., Ushijima N., Hida Y., Sano H., Kitagawa Y. (2022). Antiviral effect of cetylpyridinium chloride in mouthwash on SARS-CoV-2. Sci. Rep..

[2] Muñoz-Basagoiti J., Perez-Zsolt D., León R., Blanc V., Raïch-Regué D., Cano-Sarabia M., Trinité B., Pradenas E., Blanco J., Gispert J. (2021). Mouthwashes with CPC reduce the infectivity of SARS-CoV-2 variants in vitro. J. Dent. Res..

[3] Anderson E.R., Patterson E.I., Richards S., Pitol A.K., Edwards T., Wooding D., Buist K., Green A., Mukherjee S., Hoptroff M. (2022). CPC-containing oral rinses inactivate SARS-CoV-2 variants and are active in the presence of human saliva. J. Med. Microbiol..

[4] Seneviratne C.J., Balan P., Ko K.K.K., Udawatte N.S., Lai D., Ng D.H.L., Venkatachalam I., Lim K.S., Ling M.L., Oon L. (2021). Efficacy of commercial mouth-rinses on SARS-CoV-2 viral load in saliva: Randomized control trial in Singapore. Infection.

[5] Eduardo F.P., Corrêa L., Heller D., Daep C.A., Benitez C., Malheiros Z., Stewart B., Ryan M., Machado C.M., Hamerschlak N. (2021). Salivary SARS-CoV-2 load reduction with mouthwash use: A randomized pilot clinical trial. Heliyon.

[6] Shibata T., Konishi K. (2020). The respiratory chain of bacteria is a target of the disinfectant MA-T. BPB Rep..

[7] Hatanaka N., Xu B., Yasugi M., Morino H., Tagishi H., Miura T., Shibata T., Yamasaki S. (2021). Chlorine dioxide is a more potent antiviral agent against SARS-CoV-2 than sodium hypochlorite. J. Hosp. Infect..

[8] Chan A.W., Tetzlaff J.M., Gøtzsche P.C., Altman D.G., Mann H., Berlin J.A., Dickersin K., Hróbjartsson A., Schulz K.F., Parulekar W.R. (2013). SPIRIT 2013 explanation and elaboration: Guidance for protocols of clinical trials. BMJ.

[9] Cárdenas A.M., Campos-Bijit V., Di Francesco F., Schwarz F., Cafferata E.A., Vernal R. (2022). Electrolyzed water for the microbiologic control in the pandemic dental setting: A systematic review. BMC Oral Health.

